# Effects of Eight Weeks of Aerobic Training Combined with Carbohydrate Mouth Rinse on Body Composition and Exercise Performance in Adult Men with Obesity: Evidence from Korea

**DOI:** 10.3390/metabo15070455

**Published:** 2025-07-05

**Authors:** Jae-Myun Ko, Wi-Young So, Sung-Eun Park

**Affiliations:** 1 Department of Physical Education, Yonsei University, Seoul 03722, Republic of Korea; kjm1218@yonsei.ac.kr; 2 Department of Sports Medicine, College of Humanities, Korea National University of Transportation, Chungju-si 27469, Republic of Korea; 3 Department of Sport Science, University of Seoul, Seoul 02504, Republic of Korea

**Keywords:** carbohydrate, mouthwashes, exercise, aerobic, physiological phenomena

## Abstract

**Background:** Considering that the prevalence of obesity has risen rapidly in recent decades, the aim of this study was to investigate the effects of a carbohydrate mouth rinse (CMR) on the outcomes of aerobic training among adult men with obesity, focusing particularly on the effects of repeated use on body composition and exercise performance. **Methods:** The intervention targeted 20 men with obesity in their 20s and 30s randomly assigned to either a CMR group (n = 10) or a placebo mouth rinse (PMR) group (n = 10). Both groups completed treadmill-based aerobic training three times per week for eight weeks. Prior to each session, participants used a mouth rinse at 60, 40, and 20 s before the start of each exercise, holding either a 6% maltodextrin solution (CMR) or purified water (PMR) in their mouths for 5 to 10 s before expectorating. Pre- and post-intervention assessments included body composition (body weight and body fat percentage), resting metabolic rate (RMR), maximal oxygen uptake (VO_2_max), and exercise performance (rate of perceived exertion [RPE], exercise distance, speed, and time). Data were analyzed using 2 × 2 repeated measures analysis of variance. **Results:** Following the intervention, the CMR group showed significantly greater improvements than the PMR group did in body fat percentage, RMR, VO_2_max, exercise distance, speed, and time (*p* < 0.01). However, the interaction effect for RPE was not statistically significant between the groups (*p* = 0.175). Overall, the repeated use of the CMR during aerobic training contributed to enhanced exercise performance and favorable physiological changes without additional caloric intake. **Conclusions:** A CMR may be a practical and non-caloric ergogenic aid to support exercise performance and metabolic function in individuals with obesity. Its repeated use during aerobic training appears to be effective and safe, especially when fasting while exercising, when improving endurance without compromising fat loss is essential.

## 1. Introduction

Carbohydrates are an important energy source used by the human body during exercise. Their relative contribution to energy metabolism increases with longer exercise duration and greater exercise intensity, especially from moderate intensity onward [1]. In 1920, Krogh and Lindhard [2] first reported that a high-carbohydrate diet could lessen exercise-induced fatigue. Subsequent studies have confirmed that carbohydrate intake when exercising at maximal oxygen uptake (VO_2_max) of 70–75% helps prolong one’s endurance [3,4,5]. Thus, the American College of Sports Medicine recommends ingesting 30 to 60 g of carbohydrates during exercise [6], noting that supplementing carbohydrates not only spares protein but also directly enhances exercise performance via effects on the central nervous system [7]. However, the oral ingestion of carbohydrates also stimulates insulin secretion, leading to metabolic alterations and potentially causing gastrointestinal discomfort, which can negatively affect exercise performance [8,9,10]. The carbohydrate mouth rinse (CMR) has been developed for these reasons; rather than swallowing a solution, a small amount of carbohydrate-containing liquid is held in the mouth and then expectorated. This approach minimizes gastrointestinal load and takes advantage of the relatively high permeability of the oral mucosa, allowing for partial carbohydrate absorption [4].

In 2004, Carter et al. [11] first reported that exercise performance could be improved simply by rinsing the mouth with carbohydrates without actually swallowing them. Since then, many studies have demonstrated that a CMR can enhance performance during moderate-to-high intensity exercise lasting approximately one hour, such as cycling and running [12,13]. For example, in one-hour cycling time trials, using a CMR improved performance by approximately 2% [14], 3% [11], and 4% [7] compared with the results under the control conditions; a 30 min running time trial also reported an approximate 2% improvement with a CMR compared with that under the control conditions [15]. According to functional magnetic resonance imaging studies examining human brain activation, holding a carbohydrate solution in the mouth stimulates receptors on the tongue, activating brain regions associated with taste and reward, such as the insula/anterior cingulate cortex, orbitofrontal cortex, and striatum. Notably, artificial sweeteners do not produce these effects [4,14]. In other words, there appears to be a specific pathway for sensing carbohydrate molecules, and simply providing a sweet taste with non-nutritive sweeteners does not elicit the same central effects [14,16]. Although we have yet to fully clarify the exact mechanisms underlying the CMR, there is growing evidence indicating that a CMR can reduce the negative effects associated with oral carbohydrate ingestion, such as gastrointestinal discomfort, while activating specific brain regions. Thus, the CMR is considered a useful strategy for improving exercise performance without additional caloric intake.

However, the effects of a CMR may depend on the nutritional status of the person’s body prior to exercise, particularly the availability of carbohydrate stores [13,17,18]. When endogenous carbohydrate stores are sufficient, the mouth rinse benefit may be minimal; conversely, when carbohydrate availability is low, such as during fasting or in a fasting state, the mouth rinse may significantly improve exercise performance [13,19,20]. This suggests that a CMR could be particularly effective for individuals engaging in calorie restriction or fasting for weight loss purposes. Nevertheless, most studies have evaluated the effects of the mouth rinse in healthy athletes or trained adults, primarily examining its acute, single-use effects. Medium- to long-term studies of the use of a CMR in individuals with obesity undergoing dietary control and exercise for fat loss remain scarce.

Therefore, the primary objective of this study was to examine the effects of combining eight weeks of moderate-intensity aerobic exercise with a CMR on body composition in adult men with obesity engaged in dietary restriction for weight loss. In addition, this study investigated its effects on aerobic performance (e.g., VO_2_max and time to exhaustion) and resting metabolic rate. Based on prior findings, we hypothesized that combining a CMR with aerobic training would lead to greater improvements in body composition, aerobic capacity, and resting metabolic rate than aerobic training alone.

Given the steadily increasing rates of obesity worldwide and the limited long-term success of conventional interventions, there is a clear need for innovative and practical strategies to enhance exercise performance and training adherence in this population. Through this research, we expect to propose a novel approach to improving exercise performance and training efficiency in populations with obesity by confirming the effectiveness and practical significance of the CMR as a strategy applicable in real-world settings.

## 2. Methods

### 2.1. Participants

Our participants were adult men with obesity aged 20 to 30 years in Korea, with no medical conditions and smoking or drinking habits and who had not engaged in regular exercise in the preceding three months. All participants met the obesity criteria of BMI ≥ 25 kg/m^2^ and body fat percentage ≥ 25%. After receiving a full explanation of the study’s purpose and procedures, all participants provided written informed consent. We conducted our initial eligibility screening via preliminary interviews and questionnaires. Ultimately, we selected 20 eligible participants and randomly assigned them to either the CMR group (CMR, n = 10) or the placebo mouth rinse group (PMR, n = 10) prior to the intervention. Although no standardized dietary protocol was applied, the participants were instructed to refrain from alcohol consumption, smoking, and late-night eating in order to minimize lifestyle-related confounding variables. All exercise sessions were conducted in a fasted state (i.e., after overnight fasting), and the participants were advised to maintain consistent lifestyle habits during the 8-week period.

We calculated the required sample size using G*Power software (version 3.1.7; Heinrich-Heine-University, Düsseldorf, Germany) for a 2 × 2 repeated measures analysis of variance (ANOVA). Body fat percentage was selected as the primary outcome variable for this calculation, because it is a key indicator of body composition change and has been frequently used in previous studies evaluating the effects of aerobic training. Assuming an effect size of 0.40, an alpha level of 0.05, and a desired power (1-β) of 0.80, the minimum number of participants required was 16. To account for potential dropout, we recruited a total of 20 participants, ensuring sufficient statistical power for the analyses.

In addition, we employed a single-blind design, with participants unaware of their group allocation and the nature of the solutions used. The Institutional Review Board of Yonsei University approved our study (approval number: 7001988-201708-HR-248-02, approval date: 31 August 2017), which was conducted in accordance with the ethical principles of the Declaration of Helsinki. All participants were informed about the purpose of study and voluntarily signed an informed consent form.

### 2.2. Experimental Design and Procedures

We employed a repeated measures design, in which all participants completed an eight-week aerobic training program and underwent pre- and post-intervention assessments for the same set of outcome variables. Pre- and post-intervention assessments were conducted in the exercise prescription room of the fitness center at Y University. The participants were instructed in advance to avoid excessive physical activity or alcohol consumption the day before testing and to maintain a fasting state for at least 12 h prior to each assessment. To ensure physiological stability, the order of the measurements was as follows: RMR, body composition, VO_2_max, and exercise performance. This study employed a parallel-group randomized controlled design, in which each participant was exposed exclusively to either the CMR or PMR condition throughout the study period. Therefore, there were no carryover or sequence effects inherent to crossover designs.

### 2.3. Interventions

#### 2.3.1. Aerobic Exercise Program

The CMR and PMR groups participated in the same eight-week aerobic training program, performing supervised exercise sessions three times per week at the university fitness center. The exercise consisted of running on a treadmill, with the target exercise intensity individually set at 75% of each participant’s VO_2_max, as determined by a preliminary graded exercise test. Specifically, we calculated the target heart rate (HR) using the Karvonen formula and frequently adjusted the treadmill speed and incline to maintain the participant’s heart rate near the prescribed target during exercise. To minimize psychological bias, real-time feedback regarding exercise distance, speed, and time was blinded from the participants. Prior to the intervention, all participants completed a two- to three-day orientation and treadmill familiarization period to ensure they could adapt to the testing environment. Each training session lasted approximately 30 min; according to the principle of progressive overload, the duration gradually increased from an initial 20 min to up to 40 min by the end of the program, with the rate of progression tailored to individual fitness levels.

Each session included a 5 min warm-up and a 5 min cool-down period consisting of low-intensity walking. The exercise sessions were conducted individually, not in group format, and scheduled consistently in the morning (between 7:00 and 9:00 a.m.) for all participants. No active or passive rest periods were provided during the treadmill exercise, which was continuous.

#### 2.3.2. Carbohydrate Mouth Rinse Treatment

The CMR group used a 6% maltodextrin solution (MyProtein, Manchester, UK) prior to each exercise session, while the PMR group used an identical rinse protocol with purified water and no nutritional content. The carbohydrate solution was prepared by dissolving accurately weighed maltodextrin (measured with a SCALTEC SBC-31, Germany electronic balance) in purified water to a total volume of 25 mL. The solution was placed in 50 mL beakers and provided to the participants before exercise. The mouth rinse protocol was based on previous studies [21], and to maximize effectiveness, the participants used the rinse at three time points: 60 s, 40 s, and 20 s before starting to exercise. At each time point, the participants held the solution in their mouths for approximately 5 to 10 s before expectorating into a dedicated disposal container, ensuring that no solution was swallowed. All procedures were strictly supervised by study personnel. We designed the study as a single-blind protocol to ensure that the participants were unaware of their group allocation or the nature of the solution, thereby minimizing psychological expectancy effects. Although the PMR condition involved rinsing with purified water, it provided no nutritional or physiological effects. Thus, from a physiological viewpoint, the PMR condition can be considered equivalent to aerobic training alone.

### 2.4. Measurements

To ensure consistency and minimize inter-rater variability, all measurements—including body composition, RMR, VO_2_max, and exercise performance—were conducted by the same trained examiner throughout the study. The assessor was a certified exercise physiologist with substantial experience in human performance and metabolic testing. All assessments followed standardized protocols and were conducted under identical environmental conditions to ensure reliability.

#### 2.4.1. Body Composition

We assessed body composition with all participants in a fasted and rested state, following a minimum of 12 h of overnight fasting. Height was measured to the nearest 0.1 cm using an automatic stadiometer (Fanics FE-810, Seoul, Republic of Korea), while body weight and body fat percentage were determined using a bioelectrical impedance analyzer (InBody 720, Biospace, Seoul, Republic of Korea). To ensure measurement accuracy, we instructed the participants to void their bladder and bowels immediately prior to testing, remove all metal objects (such as accessories and watches), and wear light clothing. During the measurements, the participants were guided to maintain correct posture and a relaxed state to ensure optimal electrode contact.

#### 2.4.2. RMR

All participants were instructed to refrain from excessive physical activity for 48 h prior to measurement and to obtain at least 7 h of sleep, arriving at the Exercise Physiology Laboratory of Yonsei University with minimal activity by 6:00 the next morning. This protocol was designed to minimize the physiological effects of recent physical activity, such as excess post-exercise oxygen consumption (EPOC), which can influence resting metabolic rate (RMR) measurements. For the pre-test, the RMR was sequentially measured starting 2 weeks prior to anthropometric assessment, blood sampling, and the graded exercise test. The same procedures were followed for the post-test, with the RMR measured sequentially during the 2 weeks following the completion of the 8-week aerobic training intervention. During all RMR assessments, the participants were measured using the Quark RMR Canopy Hood (Cosmed, Rome, Italy), which enclosed the area from the head to below the chest to prevent external air from entering. The participants were instructed to breathe comfortably and naturally throughout the measurement and were monitored to ensure they did not fall asleep. Subsequently, their resting metabolic rate was measured using the breath-by-breath method with the Canopy System of the Metabolic Measurement System (TrueOne 2400, ParvoMedics, Murray, UT, USA). The stability of breathing was confirmed by monitoring the respiratory exchange ratio (R), and the flowmeter fan speed was adjusted as needed. Each session lasted approximately 30 to 40 min, with the initial 10 min considered an adaptation period and excluded from analysis. The mean value from the remaining 20 min was used for data analysis.

#### 2.4.3. VO_2_max

Maximal oxygen uptake (VO_2_max) was measured in the exercise prescription room of the fitness center at Y University. After anthropometric and body composition assessments, all participants rested for more than 10 min until their heart rates reached a stable resting level before testing commenced. Each participant wore a wireless heart rate monitor (FT2, Polar, Finland) and performed a graded exercise test on a treadmill (TM65 Treadmill, Quinton, Washington, DC, USA) with respiratory gas analysis using a metabolic measurement system (TrueOne 2400, ParvoMedics, Murray, UT, USA). Considering that the participants were individuals with obesity without prior experience in fitness testing, the Modified Bruce Treadmill Max Protocol was employed to ensure safe assessment of maximal oxygen uptake (VO_2_max).

#### 2.4.4. Exercise Performance

To objectively assess exercise performance, the distance (m), speed (km/h), and time (min) achieved during each session were recorded based on the final readings displayed on the treadmill monitor. The participants wore a Polar heart rate monitor during training, and their heart rate and rate of perceived exertion (RPE) were recorded every 3 min by a supervisor. The participants’ RPE was assessed using the Borg scale (6–20 points), and the maximum value was used for analysis. All measurements were conducted under the supervision of trained examiners in the same environment throughout the 8-week intervention period. Importantly, to ensure objectivity of the exercise performance data, the participants were blinded their performance indicators (distance, speed, or time) during the exercise sessions by covering the treadmill display.

### 2.5. Statistical Analysis

All data are presented as means ± standard deviations. To examine within-group (pre- vs. post-intervention) and between-group (CMR vs. PMR) differences, we conducted a 2 × 2 repeated measures ANOVA (group: CMR vs. PMR × time: pre vs. post). When a significant group × time interaction was identified, simple effects analyses were performed to further investigate the nature of the interaction. Specifically, paired *t*-tests were used to assess within-group changes over time, and independent *t*-tests were used to evaluate between-group differences at each time point. To control for Type I errors due to multiple comparisons, Bonferroni correction was applied to the significance level for all pairwise comparisons. We used SPSS software (version 26.0; IBM Corp., Armonk, NY, USA) for all our statistical analyses, with statistical significance set at α = 0.05 (Bonferroni-adjusted as appropriate).

## 3. Results

### 3.1. Characteristics of the Participants

Before presenting the main outcomes, we confirmed that there were no significant differences between the two groups in baseline characteristics. These are summarized in Table 1.

### 3.2. Changes in Body Composition and Resting Metabolic Rate

After the eight-week aerobic training intervention, the CMR and PMR groups showed a lower body weight and body fat percentage, along with a slight increase in the RMR (Table 2). In the CMR group, body weight decreased from 89.93 ± 8.25 kg at baseline to 81.81 ± 5.59 kg post-intervention, whereas the PMR group decreased from 91.60 ± 4.65 kg to 86.10 ± 3.83 kg. However, the group × time interaction effect was not statistically significant (F[1,18] = 3.74, *p* = 0.069). The body fat percentage decreased from 33.86 ± 2.85% to 27.68 ± 2.30% in the CMR group and from 34.62 ± 2.83% to 30.96 ± 2.25% in the PMR group, with a statistically significant group × time interaction effect (F[1,18] = 12.25, *p* = 0.003). Lean body mass increased slightly in the CMR group (from 36.27 ± 2.00 kg to 37.05 ± 1.65 kg) and decreased in the PMR group (from 35.84 ± 1.87 kg to 35.28 ± 2.45 kg), showing a significant group × time interaction (F[1,18] = 8.13, *p* = 0.011). The RMR increased from 1689.67 ± 81.14 kcal/day to 1743.35 ± 95.51 kcal/day in the CMR group and from 1698.56 ± 63.22 kcal/day to 1722.96 ± 63.21 kcal/day in the PMR group. The group × time interaction for RMR was also statistically significant (F[1,18] = 11.64, *p* = 0.003).

Values are presented as means ± standard deviations. Within-group (pre vs. post) comparisons were performed using paired *t*-tests with Bonferroni correction. Between-group comparisons at each time point were performed using independent *t*-tests with Bonferroni correction. Two-way repeated measures ANOVA was used to test main and interaction effects. * *p* < 0.05, ** *p* < 0.01, and *** *p* < 0.001 (Bonferroni-adjusted for multiple comparisons). CMR, carbohydrate mouth rinse; PMR, placebo mouth rinse; CV, coefficient of variation; RMR, resting metabolic rate.

### 3.3. Changes in Exercise Performance

As shown in Table 3, the RPE decreased from 16.30 ± 0.95 to 14.00 ± 1.76 in the CMR group and from 16.50 ± 1.08 to 15.10 ± 1.20 in the PMR group. However, the group × time interaction effect was not statistically significant (F[1,18] = 2.00, *p* = 0.175). VO_2_max increased from 33.42 ± 2.23 to 38.65 ± 1.91 mL/kg/min in the CMR group and from 34.02 ± 1.76 to 37.32 ± 1.03 in the PMR group; the group × time interaction was statistically significant (F[1,18] = 10.13, *p* = 0.005). The exercise distance (km) increased from 4.13 ± 0.31 to 7.01 ± 0.32 in the CMR group and from 3.88 ± 0.52 to 5.07 ± 0.80 in the PMR group, again with a statistically significant group × time interaction (F[1,18] = 30.86, *p* < 0.001). Exercise speed (km/h) increased from 6.40 ± 0.55 to 8.19 ± 0.70 in the CMR group and from 6.55 ± 0.39 to 7.24 ± 0.32 in the PMR group, with another significant group × time interaction (F[1,18] = 30.44, *p* < 0.001). Finally, exercise time (min) increased from 33.35 ± 2.88 to 43.39 ± 3.02 in the CMR group and from 32.91 ± 3.80 to 38.05 ± 4.05 in the PMR group; this group × time interaction was also statistically significant (F[1,18] = 8.56, *p* = 0.009).

## 4. Discussion

Our aim was to investigate the effects of eight weeks of aerobic training combined with a CMR on body composition and exercise performance in men with obesity aged 20 to 30 years old in Korea. The results showed that the CMR group had significantly greater improvements in body fat percentage, RMR, VO_2_max, exercise distance, speed, and time compared with those in the PMR group. These differences were statistically significant, as indicated by group × time interaction effects (*p* < 0.001). Thus, while the acute effects of the CMR have been demonstrated in prior studies [11,15], our findings extend this knowledge by showing that repeated application during an eight-week training program can enhance exercise-related outcomes.

The mouth rinse protocol used involved rinsing with a carbohydrate solution for 5 to 10 s and expectorating without ingestion. This strategy is particularly suitable for fasted aerobic exercise among individuals with obesity. Indeed, we observed significant reductions in body weight and body fat percentage in both groups, but with a greater reduction in body fat percentage noted in the CMR group. These findings are consistent with the studies of Carter et al. [11] and de Ataide e Silva et al. [12], who also reported that mouth rinsing with carbohydrates enhanced performance without providing additional caloric intake. Moreover, although the changes in lean body mass were modest, the CMR group exhibited a slight increase post-intervention, while the PMR group showed a slight decrease. Given that lean mass is a key determinant of resting metabolic rate [22], this preservation of lean mass may partially explain the greater metabolic improvement observed in the CMR group. One plausible explanation is that reduced perceived exertion in the CMR group helped maintain higher-quality training sessions, enabling better muscle engagement and recovery, even under fasting conditions [23,24].

Similarly, the RMR increased significantly in the CMR group (F[1,18] = 11.64, *p* = 0.003). RMR is known to be influenced by lean body mass [22], and increases in muscle mass or improvements in oxidative metabolic efficiency as a result of training can lead to higher RMR values [23,25]. In individuals with obesity, basal metabolic efficiency is often impaired [24], but such metabolic characteristics can be gradually improved through regular aerobic exercise training [26,27,28]. In particular, exercise performed repeatedly while fasting in this study may have contributed to metabolic adaptations such as enhanced fat utilization and increased energy expenditure; however, these interpretations remain speculative, as direct assessments of substrate oxidation and daily energy balance were not conducted. This is consistent with the findings of Vieira et al. [8], who reported that fasting during aerobic exercise enhances fat loss and lipid metabolism.

Notably, the CMR intervention contributed to a significant increase in RMR without any actual additional caloric intake. This may have been partially related to a trend toward reduced subjective fatigue in the CMR group, although the group × time interaction for RPE was not statistically significant. This non-significant tendency might have helped participants sustain consistent training quality, potentially contributing to the overall metabolic stimulus of the intervention. In addition, repeated use of the CMR may have amplified the cumulative training load by improving adherence and perceived effort, thereby increasing total energy turnover and post-exercise oxygen consumption, which are key contributors to an elevated RMR. These effects may also relate to all the exercise sessions being performed while fasting. Previous studies have reported that the effectiveness of a CMR may differ depending on pre-exercise energy status (fasted vs. fed) [13,20]. In our study, as all participants exercised in the morning after an overnight fast, the central nervous system may have been more sensitive to the intervention. Vieira et al. [8] reported that exercise while fasting increases fat oxidation and minimizes blood glucose fluctuations, and such physiological mechanisms likely contributed to the positive metabolic effects of the CMR we observed in this study, without impeding weight loss.

In terms of cardiorespiratory fitness, the VO_2_max in the CMR group increased significantly from the baseline (F[1,18] = 10.13, *p* = 0.005), with this improvement being more pronounced compared with that in the PMR group, despite both groups undergoing the same intensity training. In general, increases in the VO_2_max reflect improvements in oxygen delivery systems (such as cardiac output and blood flow distribution) and oxygen utilization systems (such as mitochondrial function in muscle cells) [29,30]. Given that all exercise sessions were performed in a fasted state, it is possible that the enhanced central drive associated with the CMR—through activation of oral carbohydrate receptors—may have supported greater exercise effort and consistency during training, thereby amplifying physiological adaptations related to aerobic capacity. This suggests that the CMR may have contributed not only to improved exercise performance but also to additional physiological changes induced by the training program.

In terms of the exercise performance indicators, such as distance, speed, and time, the CMR group showed significantly greater improvements than the PMR group did, consistent with previous findings on the effects of a CMR. For example, Rollo et al. [15] reported that using a CMR significantly increased distance and exercise time during a 30 min treadmill running trial. Similarly, a meta-analysis by Brietzke et al. [31] found that a CMR produced significant effects in endurance exercise performance time trials. Notably, in our study, the beneficial effects of the CMR were sustained over the eight-week training period, with consistent improvements observed in exercise distance, time, and speed. Although tolerance or diminished effects with repeated use are sometimes reported for centrally acting ergogenic aids [32], our results suggest that the repeated pre-training mouth rinse did not diminish efficacy. This implies that a CMR is not only effective as an acute intervention but also as a practical strategy to maximize cumulative training benefits over the long term.

The improvements in exercise performance are closely related to the effect of the CMR on lowering the RPE during exercise. We found that the CMR group showed a somewhat lower RPE compared with that in the PMR group under the same conditions, although the group × time interaction was not statistically significant. Although our findings are partly consistent with the established mechanism whereby the activation of oral carbohydrate receptors leads to central nervous system stimulation and reduced fatigue, further research is needed to clarify this effect. Chambers et al. [14] demonstrated, via functional magnetic resonance imaging, that the detection of carbohydrates in the oral cavity activates brain regions involved in reward and motor control, such as the insula, orbitofrontal cortex, and striatum. Turner et al. [33] also reported that a CMR activates the primary motor cortex and sensory cortex, thereby contributing to enhanced exercise performance. Interestingly, previous studies using artificial sweeteners such as saccharin or other placebo solutions found that these did not produce similar brain responses, indicating the presence of specific carbohydrate receptors and a non-metabolic signaling pathway [14,31]. In contrast, our study used pure water as the placebo mouth rinse solution. In other words, even without ingestion, the mere presence of carbohydrates in the mouth can stimulate the central nervous system, thereby enhancing motivation and reducing fatigue, which, in turn, may help maintain consistent training intensity and volume across sessions. Over time, this sustained training quality may accumulate to produce greater exercise adaptations, such as improvements in endurance, energy expenditure, and metabolic efficiency. These mechanisms offer a plausible physiological explanation for the observed reductions in RPE and the longer exercise endurance [14,15,33].

In sum, we demonstrated that the CMR was effective when consistently applied before training sessions. Although our study did not directly assess the acute effects of a single CMR application, the significant improvements observed over the 8-week training period suggest that repeated use may lead to meaningful physiological adaptations. Given these findings, the CMR appears to be a practical strategy for enhancing exercise performance while minimizing caloric intake, particularly in exercise settings combined with weight management. Notably, long-term CMR use was associated with improvements in resting metabolic rate and cardiorespiratory fitness, suggesting both central (cognitive) and peripheral (metabolic and circulatory) benefits.

However, our study also had some limitations. First, we limited the participants to men with obesity in their 20s and 30s in Korea, which restricted the generalizability of the findings; future research should include a broader range of ages and both sexes. Second, although all participants were instructed to refrain from alcohol consumption, smoking, and late-night eating during the intervention period, no standardized dietary protocol was applied, and daily dietary intake and energy expenditure was not quantitatively monitored or controlled. Nonetheless, the significant improvements observed in body composition and resting metabolic rate suggest that the combination of a CMR and aerobic exercise exerted a meaningful effect. Future studies should consider implementing more systematic dietary control or standardized nutritional protocols and objective assessments of energy balance to more clearly determine the effectiveness of a carbohydrate mouth rinse in individuals with obesity. Third, this study is limited by the fact that the outcome measures were primarily confined to body composition and aerobic capacity. Future research should include a more diverse range of participants and assess various physiological and psychological variables, such as metabolism, fatigue, and safety, to more clearly establish the effects and clinical utility of a CMR.

Nevertheless, this study is among the first to examine the effects of a CMR in individuals with obesity and to demonstrate that a CMR can enhance exercise performance even in a fasting state. Furthermore, our findings provide scientific evidence supporting the beneficial effects of oral rinsing interventions in aerobic exercise strategies for individuals with obesity, suggesting that a CMR can be a safer and more practical strategy for obesity management.

## 5. Conclusions

This study demonstrated that the repeated application of a CMR combined with aerobic training over eight weeks led to significant improvements in body composition, resting metabolic rate, and aerobic capacity in men with obesity. These findings support the use of a CMR as a practical, non-caloric ergogenic strategy for enhancing exercise performance and metabolic function, particularly in populations aiming for weight reduction. Given its ease of application and the observed trend toward lower perceived exertion, a CMR may serve as a useful adjunct to structured exercise programs for individuals with obesity. Future studies should explore sex-based differences and long-term safety and efficacy in more diverse populations.

## Figures and Tables

**Table 1:** Characteristics of the participants.

Characteristic	Group		*p*
	CMR (n = 10)	PMR (n = 10)	
Age (years)	27.80 ± 2.25	29.90 ± 3.07	0.100
Height (cm)	172.60 ± 3.57	175.02 ± 3.87	0.163
Weight (kg)	89.93 ± 8.25	91.60 ± 4.65	0.584
Body fat (%)	33.86 ± 2.85	34.62 ± 2.83	0.557
Lean body mass (kg)	36.27 ± 2.00	35.84 ± 1.87	0.625
BMI (kg/m^2^)	30.16 ± 2.18	29.92 ± 1.64	0.790
VO_2_max (mL/kg/min)	33.42 ± 2.23	34.02 ± 1.76	0.512

Values are presented as mean ± standard deviations; tested using independent *t*-tests. CMR, carbohydrate mouth rinse; PMR, placebo mouth rinse.

**Table 2:** Changes in body composition and resting metabolic rate before and after aerobic training intervention accompanied by rinsing.

Variable	CMR (n = 10)				Within Group	PMR (n = 10)				Within Group	Between Group	Group × Time	
	Pre	CV	Post	CV	*p*	Pre	CV	Post	CV	*p*	*p* (Pre/Post)	F	*p*
Weight (kg)	89.93 ± 8.25	0.09	81.81 ± 5.59	0.07	<0.001 ***	91.60 ± 4.65	0.05	86.10 ± 3.83	0.04	0.015 *	0.501 0.046 *	3.74	0.069
Body fat percentage (%)	33.86 ± 2.85	0.08	27.68 ± 2.30	0.08	<0.001 ***	34.62 ± 2.83	0.08	30.96 ± 2.25	0.07	<0.001 ***	0.537 0.038 *	12.25	0.003 **
Lean body mass (kg)	36.27 ± 2.00	0.06	37.05 ± 1.65	0.04	0.116	35.84 ± 1.87	0.05	35.28 ± 2.45	0.07	0.320	0.583 0.093	8.13	0.011 *
RMR (kcal/day)	1689.67 ± 81.14	0.05	1743.35 ± 95.51	0.06	0.002 **	1698.56 ± 63.22	0.04	1722.96 ± 63.21	0.04	0.008 **	0.774 0.446	11.64	0.003 **

**Table 3:** Changes in exercise performance (RPE, VO_2_max, exercise distance, speed, and time) before and after aerobic training intervention accompanied by rinsing.

Variable	CMR (n = 10)				Within Group	PMR (n = 10)				Within Group	Between Group	Group × Time	
	Pre	CV	Post	CV	*p*	Pre	CV	Post	CV	*p*	*p* (Pre/Post)	F	*p*
RPE	16.30 ± 0.95	0.06	14.00 ± 1.76	0.13	<0.001 ***	16.50 ± 1.08	0.07	15.10 ± 1.20	0.08	0.002 **	0.655 0.046 *	2.00	0.175
VO_2_max (mL/kg/min)	33.42 ± 2.23	0.07	38.65 ± 1.91	0.05	<0.001 ***	34.02 ± 1.76	0.05	37.32 ± 1.03	0.03	<0.001 ***	0.443 0.093	10.13	0.005 **
Exercise distance (km)	4.13 ± 0.31	0.08	7.01 ± 0.32	0.05	<0.001 ***	3.88 ± 0.52	0.13	5.07 ± 0.80	0.16	<0.001 ***	0.285 <0.001 ***	30.86	<0.001 ***
Exercise speed (km/h)	6.40 ± 0.55	0.09	8.19 ± 0.70	0.09	<0.001 ***	6.55 ± 0.39	0.06	7.24 ± 0.32	0.04	<0.001 ***	0.589 0.002 *	30.44	<0.001 ***
Exercise time (min)	33.35 ± 2.88	0.09	43.39 ± 3.02	0.07	<0.001 ***	32.91 ± 3.80	0.12	38.05 ± 4.05	0.11	<0.001 ***	0.767 0.015 *	8.56	0.009 **

Values are presented as means ± standard deviations. Within-group (pre vs. post) comparisons were performed using paired *t*-tests with Bonferroni correction. Between-group comparisons at each time point were performed using independent *t*-tests with Bonferroni correction. Two-way repeated measures ANOVA was used to test main and interaction effects. * *p* < 0.05, ** *p* < 0.01, and *** *p* < 0.001 (Bonferroni-adjusted for multiple comparisons). CMR, carbohydrate mouth rinse; PMR, placebo mouth rinse; CV, coefficient of variation; RPE, rate of perceived exertion.

## Data Availability

The data supporting the findings of this study are available from the corresponding author upon request.
